# Supplementary material for: Qualitative and Quantitative Comparison of Plasma Exosomes from Neonates and Adults
Source: Int J Mol Sci. 2021 Feb 15;22(4):1926. doi: 10.3390/ijms22041926 (PMC7919666; doi:10.3390/ijms22041926)
Supplement: Supplementary file 1 [file ijms-22-01926-s001.pdf]

International Journal of
Molecular Sciences

MDPI

			Size (d.nm):	% Volume:	St Dev (d.nm):
Z-Average (d.nm): 41,83		Peak 1:	17,20	99,4	8,230
PdI: 0,550		Peak 2:	756,7	0,5	367,7
Intercept: 0,920		Peak 3:	5014	0,2	880,7
Result quality : Good					

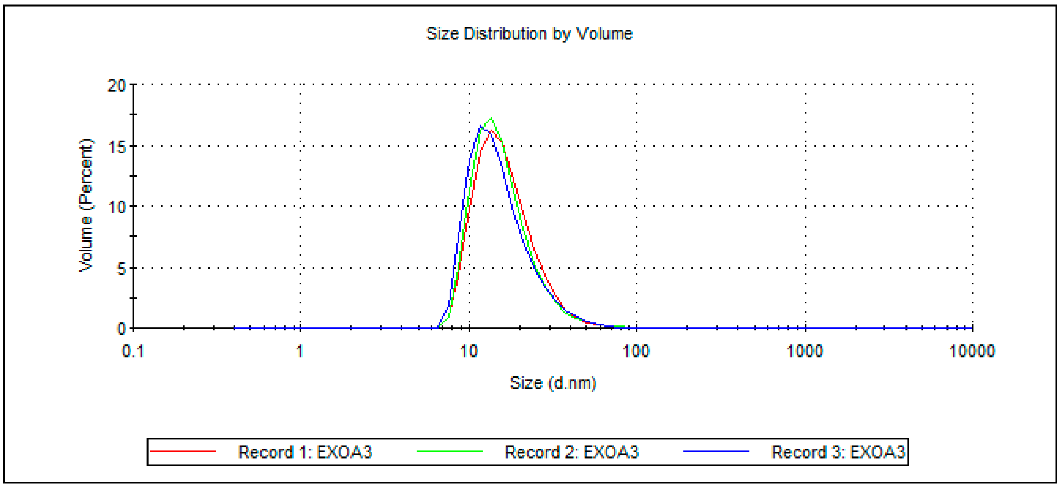

Supplementary Figure 1. Estimation of the percentage of volume of each population of extracellular vesicles by Dynamic light scattering. Each line represents an independent run.

Supplementary Table 1. Differential protein expressionidentified by SWATH MS in neonatalexosomescompared to adultsexosomes ($p < 0.05$; FCh≠1). Ig: Immunoglobulin.

Uniprot Name	Gene Name	Protein Name	*p* value	Fold Change
P02786	TFR1	Transferrin receptor protein 1	0.00104	15.32
Q99808	S29A1	Equilibrative nucleide transporter 1	0.01424	14.01
P11166	GTR1	Solute carrier family 2, facilitated glucose transporter member 1	0.00106	10.15
P69892	HBG2	Hemoglobin subunit γ-2	0	9.43
P02792	FRIL	Ferritin light chain	0.00438	9.22
Q16819	MEP1A	Meprin A subunitα	0.01481	7.13
P02776	PLF4	Platelet factor 4	0.00809	7.01
Q02094	RHAG	Ammonium transporter Rh type A	0.00772	6.71
P27918	PROP	Properdin	0	6.26
P16671	CD36	Platelet glycoprotein 4	0.00001	5.99
Q9NQ84	GPC5C	G-protein coupled receptor family C group 5 member C	0.00467	5.87
P08195	4F2	4F2 cell-surface antigen heavy chain	0.00224	5.77
Q658P3	STEA3	Metalloreductase STEAP3	0.00262	5.34
Q6UX06	OLFM4	Olfactomedin-4	0.00008	5.08
P02787	TRFE	Serotransferrin	0.00101	5.05
P08514	ITA2B	Integrin αIIb	0	4.88
P02730	B3AT	Band 3 anion transport protein	0.00334	4.71
P11142	H7C	Heat shock cognate 71 kDa protein	0.00239	4.7

Q15758	AAAT	Neutral amino acid transporter B(0)	0.00829	4.56
P05164	PERM	Myeloperoxidase	0.00003	4.46
P0DMV9	HS71B	Heat shock 70 kDa protein 1B	0.0012	4.36
P27105	STOM	Erythrocyte band 7 integral membrane protein	0.00326	4.18
P02743	SAMP	Serum amyloid P-component	0	4.15
Q13201	MMRN1	Multimerin-1	0.00228	4.1
Q15907	RB11B	Ras-related protein Rab-11B	0.00058	4.03
Q9HD89	RETN	Resistin	0.00002	3.79
P01024	CO3	Complement C3	0	3.6
Q86UX7	URP2	Fermitin family homolog 3	0	3.51
P05023	AT1A1	Sodium/potassium-transporting ATPase subunit α-1	0.00875	3.39
P18428	LBP	Lipopolysaccharide-binding protein	0.00971	3.31
P04275	VWF	von Willebrand factor	0	3.29
Q00610	CLH1	Clathrin heavy chain 1	0.00667	3.15
P01859	IGHG2	Ig heavy constant γ 2	0.0001	3.06
Q9Y490	TLN1	Talin-1	0.00002	3.02
P00451	FA8	Coagulation factor VIII	0.00055	3.01
P05106	ITB3	Integrin β3	0.00003	3
P01042	KNG1	Kininogen-1	0.00652	2.95
O15400	STX7	Syntaxin-7	0.00007	2.89
P12259	FA5	Coagulation factor V	0	2.77
P26038	MOES	Moesin	0.00065	2.74
P10643	CO7	Complement component C7	0	2.73
Q9UP52	TFR2	Transferrin receptor protein 2	0.03685	2.71
P02760	AMBP	Protein AMBP	0.00118	2.7
P11597	CETP	Cholesterylester transfer protein	0.00012	2.7
P15144	AMPN	Aminopeptidase N	0.00896	2.6
P21926	CD9	CD9 antigen	0.00006	2.59
P01861	IGHG4	Ig heavy constant γ 4	0.00009	2.58
P0C0L5	CO4B	Complement C4-B	0	2.54
Q71DI3	H32	Histone H3.2	0.0305	2.49
P05546	HEP2	Heparin cofactor 2	0.00001	2.44
P08603	CFAH	Complement factor H	0.0057	2.43
P13671	CO6	Complement component C6	0.00044	2.33
P04004	VTNC	Vitronectin	0	2.3
Q9HAV0	GBB4	Guanine nucleotide-binding protein subunit β-4	0.00045	2.27
P21333	FLNA	Filamin-A	0.00019	2.23
P02788	TRFL	Lactotransferrin	0.01132	2.23
P62834	RAP1A	Ras-related protein Rap-1A	0.00141	2.2
P01717	LV325	Ig λ variable 3-25	0.04177	2.18
P02675	FIBB	Fibrinogen β chain	0.00005	2.16
P69905	HBA	Hemoglobin subunit α	0.00317	2.15
P63104	1433Z	14-3-3 protein ζ/δ	0.00005	2.14
P02679	FIBG	Fibrinogen γ chain	0	2.13
P01857	IGHG1	Ig heavy constant γ 1	0.00004	2.1
P01023	A2MG	A-2-macroglobulin	0.00104	2.02

P35579	MYH9	Myin-9	0.0129	1.98
P60709	ACTB	Actin, cytoplasmic 1	0.00113	1.93
O00560	SDCB1	Syntenin-1	0.00187	1.92
P00736	C1R	Complement C1r subcomponent	0.00311	1.9
P04899	GNAI2	Guanine nucleotide-binding protein G(i) subunit α-2	0.00899	1.88
P07737	PROF1	Profilin-1	0.01408	1.87
P10909	CLUS	Clusterin	0.00067	1.86
P68363	TBA1B	Tubulinα-1B chain	0.01478	1.8
Q16610	ECM1	Extracellular matrix protein 1	0.04064	1.78
P07996	T1	Thrombpondin-1	0.00001	1.73
P20073	ANXA7	Annexin A7	0.04188	1.7
P19827	ITIH1	Inter-α-trypsin inhibitor heavy chain H1	0.00028	1.67
Q03591	FHR1	Complement factor H-related protein 1	0.01503	1.67
P02671	FIBA	Fibrinogen α chain	0.00165	1.61
Q08380	LG3BP	Galectin-3-binding protein	0.00039	1.56
P19823	ITIH2	Inter-α-trypsin inhibitor heavy chain H2	0.00222	1.54
P48740	MA1	Mannan-binding lectin serine protease 1	0.00005	1.54
P01833	PIGR	Polymeric Ig receptor	0.03554	1.46
O75636	FCN3	Ficolin-3	0.00141	1.32
P05160	F13B	Coagulation factor XIII B chain	0.001	0.74
A0A0B4J1U7	HV601	Ig heavy variable 6-1	0.00377	0.69
Q14624	ITIH4	Inter-α-trypsin inhibitor heavy chain H4	0.01301	0.64
P01619	KV320	Ig κ variable 3-20	0.00045	0.64
P04114	APOB	Apolipoprotein B-100	0.02578	0.63
P02656	APOC3	Apolipoprotein C-III	0.00767	0.52
A0A0B4J1V0	HV315	Ig heavy variable 3-15	0.00015	0.52
P02647	APOA1	Apolipoprotein A-I	0	0.51
P06312	KV401	Ig κ variable 4-1	0.00234	0.48
P06310	KV230	Ig κ variable 2-30	0.00695	0.46
P07225	PROS	Vitamin K-dependent protein S	0.0011	0.42
P49721	PSB2	Proteasomes ubunit β type-2	0.01941	0.4
P01834	IGKC	Ig κconstant	0.00001	0.4
P01766	HV313	Ig heavy variable 3-13	0.00043	0.4
P0DOY3	IGLC3	Ig λ constant 3	0	0.39
A0A0C4DH69	KV109	Ig κ variable 1-9	0.00017	0.39
P04430	KV116	Ig κ variable 1-16	0.00037	0.37
B9A064	IGLL5	Ig λ-likepolypeptide 5	0.00002	0.36
P27169	PON1	Serum paraoxonase/arylesterase 1	0.00304	0.36
Q93050	VPP1	V-type proton ATPase 116 kDa subunit a isoform 1	0.01287	0.35
P01700	LV147	Ig λ variable 1-47	0	0.35
P00338	LDHA	L-lactatedehydrogenase A chain	0.01646	0.34
P04003	C4BPA	C4b-binding protein α chain	0	0.33
P01601	KVD16	Ig κ variable 1D-16	0.00931	0.32
P02766	TTHY	Transthyretin	0.01393	0.32
Q6NSI8	K1841	Uncharacterized protein KIAA1841	0.04514	0.31
Q16851	UGPA	UTP--gluce-1-phphate uridylyltransferase	0.04304	0.31
A0A0B4J1X5	HV374	Ig heavy variable 3-74	0.00052	0.31

A0A0C4DH25	KVD20	Ig κ variable 3D-20	0	0.31
P0DP03	HV335	Ig heavy variable 3-30-5	0	0.3
A0A0C4DH24	KV621	Ig κ variable 6-21	0.00154	0.28
P08519	APOA	Apolipoprotein(a)	0.00237	0.28
A2NJV5	KV229	Ig κ variable 2-29	0.00394	0.27
A0A0A0MS15	HV349	Ig heavy variable 3-49	0	0.27
A0A0C4DH68	KV224	Ig κ variable 2-24	0.00088	0.26
P01624	KV315	Ig κ variable 3-15	0.00002	0.25
P28072	PSB6	Proteasome subunit β type-6	0.02057	0.25
P04433	KV311	Ig κ variable 3-11	0.00002	0.24
P01591	IGJ	Ig J chain	0.00001	0.23
O43866	CD5L	CD5 antigen-like	0.00001	0.2
A0A0B4J1Y9	HV372	Ig heavy variable 3-72	0.00097	0.17
A0A075B6H9	LV469	Ig λ variable 4-69	0.00042	0.15
P00738	HPT	Haptoglobin	0.00134	0.13
P00739	HPTR	Haptoglobin-related protein	0	0.11
O14791	APOL1	Apolipoprotein L1	0.00002	0.08
P01877	IGHA2	Ig heavy constantα 2	0.00001	0.05
Q8TF72	SHRM3	Protein Shroom3	0.01003	0.05
P01876	IGHA1	Ig heavy constantα 1	0.00001	0.02

Ig: Immunoglobulin.

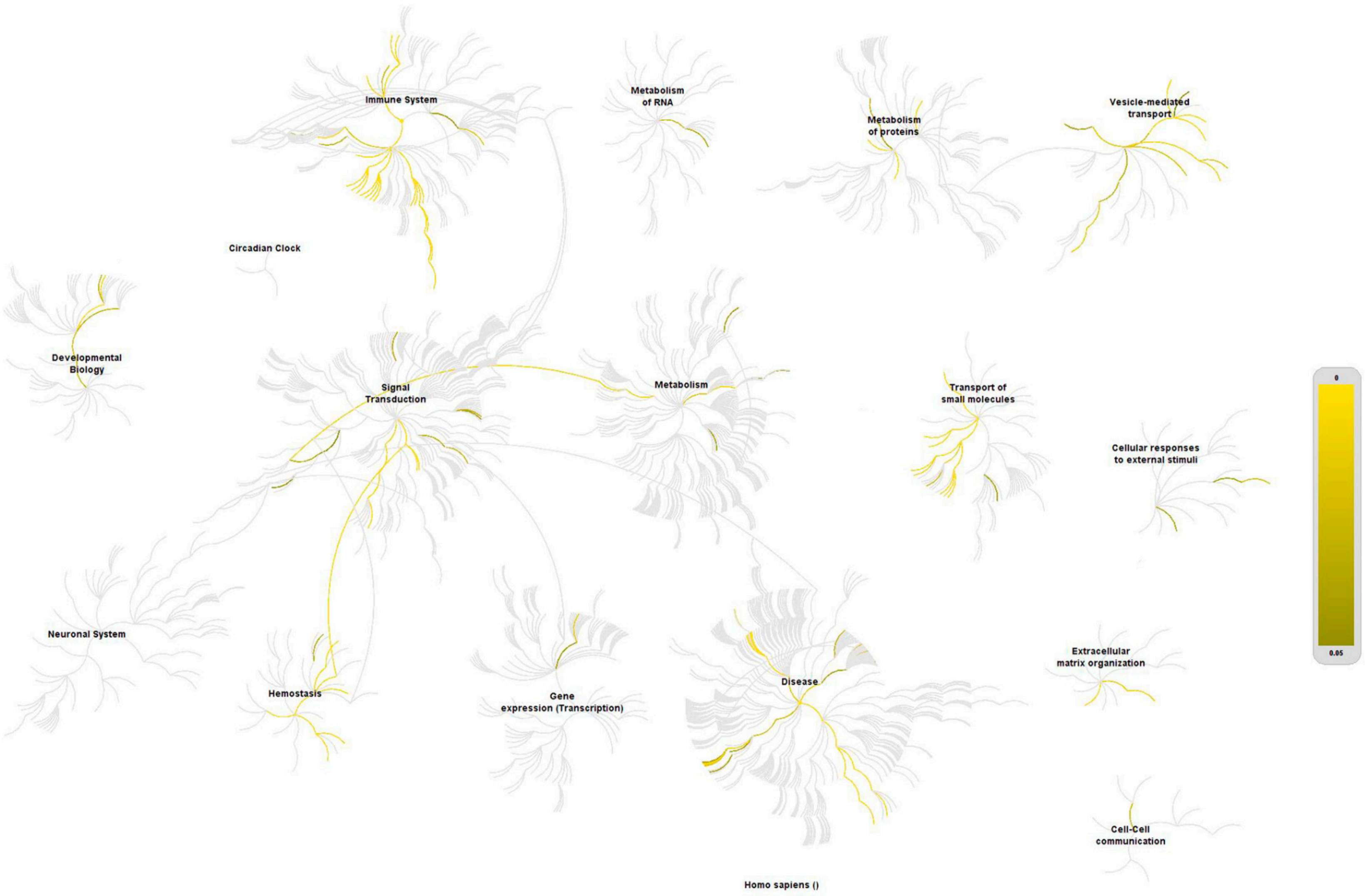

Supplementary Figure 2. Reactome network view of differentially expressed proteins. Genome-wide overview of pathway enrichment analysis. Enriched pathways are highlighted in yellow. Immune system, hemostasis, transport of small molecules, vesicle-mediated transport and developmental biology pathways cluster were the most enriched.
